# A DNA Plasmid-Based Approach for Efficient Synthesis of Sacbrood Virus Infectious Clones within Host Cells

**DOI:** 10.3390/v15091866

**Published:** 2023-09-01

**Authors:** Dandan Yue, Runlin Li, Jikailang Zhang, Yanping Chen, Evan C. Palmer-Young, Shaokang Huang, Wei-Fone Huang

**Affiliations:** 1 College of Animal Sciences (College of Bee Science), Fujian Agriculture and Forestry University, Fuzhou 350002, China; yue.dandan@outlook.com (D.Y.); runlinl@outlook.com (R.L.);; 2 Bee Research Laboratory, Agriculture Research Service, USDA, Beltsville, MD 20705, USA; 3 Honeybee Biology Observation Station, Ministry of Agriculture and Rural Affairs, Fuzhou 350002, China

**Keywords:** in-cell transcription, cDNA clones, Iflavirus, alternative hosts, cell line

## Abstract

RNA viruses are often cited as a significant factor affecting the populations of both domestic honey bees and wild pollinators. To expedite the development of effective countermeasures against these viruses, a more comprehensive understanding of virus biology necessitates extensive collaboration among scientists from diverse research fields. While the infectious virus clone is a robust tool for studying virus diseases, the current methods for synthesizing infectious clones of bee-infecting RNA viruses entail the in vitro transcription of the viral genome RNA in 8–10 kb, presenting challenges in reproducibility and distribution. This article reports on the synthesis of an infectious clone of the Chinese variant sacbrood virus (SBV) using a DNA plasmid containing an Autographa californica multiple nucleopolyhedrovirus (AcMNPV) immediate-early protein (IE1) promoter to trigger transcription of the downstream viral genome within hosts. The results demonstrate that the IE1-SBV plasmid can synthesize SBV clones in a widely used lepidopteran immortal cell line (Sf9) and honey bee pupae. Furthermore, the negative strand of the clone was detected in both Sf9 cells and honey bee pupae, indicating active infection and replication. However, the transfection of Sf9 cells was observed in only a limited proportion (less than 10%) of the cells, and the infection did not appear to spread to adjacent cells or form infective virions. The injection of honey bee pupae with 2500 ng of the IE1-SBV plasmid resulted in high infection rates in *Apis cerana* pupae but low rates in *A. mellifera* pupae, although the dosage was comparatively high compared with other studies using in vitro transcribed viral RNA. Our findings suggest that the synthesis of bee-infecting RNA viruses using DNA plasmids is feasible, albeit requiring additional optimization. However, this method holds substantial potential for facilitating the production of clones with various sequence modifications, enabling the exploration of viral gene functions and biology. The ease of distributing infectious clones in DNA plasmid form may foster collaboration among scientists in applying the clone to bee biology, ecology, and behavior, ultimately offering a comprehensive approach to managing virus diseases in the future.

## 1. Introduction

Virus diseases are considered potential culprits for the regional population decreases in honey bees [1] and wild pollinators [1,2] despite the absence of a decreasing trend in the global honey bee population [3]. In recent years, the over-winter survival rate of honey bees in the US has remained consistently low, and this decline has been attributed to viruses [4,5]. Despite the potential threats posed by viruses to honey bee health, our limited understanding of virus biology and pathogenesis presents a significant obstacle to the development of effective disease treatments and management strategies.

Most honey bee-infecting viruses documented thus far belong to the *Picornavales* order, characterized as single-stranded positive-sense RNA (+ssRNA) viruses. These viruses exhibit similarities in terms of viral capsid, genome structure, and protein translation with picornaviruses [6], which are notorious for causing severe diseases in humans, such as polio and enterovirus diseases, as well as in animals, such as foot and mouth disease in swine. Extensive research on these picornaviruses has provided in-depth insights into virus–host interactions [7,8,9], which hold the potential for developing pharmaceutical solutions [10,11,12]. However, applying the findings directly from picornavirus studies to bee-infecting RNA viruses is challenging without conducting de novo studies involving the genetic manipulation of the virus under controlled conditions. Consequently, our understanding of viral gene function and detailed virus–host interactions in honey bee-infecting RNA viruses is still relatively limited compared with the knowledge amassed for picornaviruses.

Creating infectious clones of targeted bee-infecting viruses represents the initial step in manipulating virus genomes. To date, numerous clones of bee-infecting viruses have been synthesized [13,14,15,16,17], and the use of these clones in application studies is increasingly prevalent [18,19]. As these viruses possess a single-stranded positive-sense RNA genome that mimics mRNA, the synthesized viral genome DNA templates are commonly referred to as cDNA templates. The process of editing the viral sequence to investigate gene functions is often referred to as reversed genetics [20].

However, implementing the standard method for producing infectious cloned viruses can present significant technical challenges [21]. Firstly, the need to produce full-length viral RNA in 8–10 kb sizes, which is considerably larger than the typical sizes required for gene expression or RNA interference experiments, poses a major obstacle for researchers. Moreover, RNA is susceptible to degradation, and transcribed RNA requires ultra-low temperatures during shipping. The in vitro transcription process may also be challenging to replicate in different laboratory settings, particularly those not specialized in viral or molecular biology. Furthermore, the inherent difficulties associated with replicating studies are exacerbated by these challenges. In our own research, we encountered instances where the full-length viral genome RNA of our cloned SBV [14] degraded into smaller sizes during the repetition of in vitro transcription. While quality control measures can help address this issue, they require significant time and labor resources.

Secondly, the viability of produced infectious clones may gradually diminish during storage and shipment. Currently, there is no viability assay available for bee-infecting RNA viruses, making it impossible to conduct titration assays before each inoculation. This limitation may lead to difficulties in comparing the results published using somewhat different settings. These obstacles further compound the challenges faced by laboratories involved in interdisciplinary and geographically distant collaborations when working with infectious clones.

Lastly, the requirement of inoculating viral RNA into live bees to produce infectious clones can be problematic due to the scarcity of virus-free honey bees. In addition to contaminations of the infectious clones with other viruses that have infected the testing hosts, mixed infections of wild-type and cloned viruses of the same virus species can easily deviate from the conditions necessary for generating robust and repeatable results. In our previous study of the SBV clone, we encountered difficulties due to the lack of SBV-free bees during the repeating trials [14].

The utilization of a cDNA template with self-initiating transcription capability for viral genome transcription within cultured cells offers a potential solution to the challenges associated with the use of infectious virus clones [22]. Distributing the infectious clones in DNA plasmid status would be much easier than the RNA or the purified virions. Using a DNA plasmid directly to synthesize virus clones can largely reduce difficulties, cost, and labor. Additionally, plasmids provide greater convenience for sequence editing [22]. These features would streamline the reversed genetic studies and collaborations that apply the infectious clones in various honey bee research endeavors.

In this article, we present the first attempt to create a cDNA template that can synthesize an infectious clone of a honey bee-infecting RNA virus. We used the AcMNPV immediate-early protein (IE1) promoter to generate a Chinese variant SBV clone with an EGFP expression [14]. In our previous study, this clone demonstrated infectivity and caused overt infections. AcMNPV is the most commonly used baculovirus vector in insect cell lines, and the IE1 promoter can initiate the transcription within various host cells without assistance from other viral proteins [23]. In addition to testing the IE1-SBV clone in *A. mellifera* and *A. cerana* pupae, we also evaluated its potential to infect an immortal lepidopteran cell line derived from *Spodoptera frugiperda*, known as Sf9, as an alternative host (not a natural host) for the cloned virus.

## 2. Materials and Methods

### 2.1. Ethics Statement

No commercial interests were generated from or involved in this study. All materials used in this research were properly stored and handled without being released into the field. The pGL3-IE1 plasmid was provided by Addgene (addgene.org), a non-profit repository that facilitates the sharing and distribution of plasmids among scientists. The pGL3-IE1 plasmid was obtained through their distributor in China, Beijing Zhongyuan, Ltd.

### 2.2. Plasmid Construction for Synthesis of the SBV Clone Induced by IE1 Promoter

We selected the immediate-early protein (IE1) promoter derived from the baculovirus Autographa californica multiple nucleopolyhedrovirus (AcMNPV) to drive the synthesis of the cloned virus in host cells. The IE1 promoter sequence has been extensively used for expressing various proteins in cell lines and live insects [24]. For our study, we utilized the pGL3-IE1 plasmid, which has been previously employed in live insects [25] and was generously provided by Dr. Zach Adelman (Addgene plasmid #52894). The pGL3-IE1 plasmid contains an origin of replication (pBR322) and antibiotic-resistance that matches the vector used in our previous study [14] for cloning the SBV genome, indicating feasibility of transferring the cloned SBV genome into the pGL3-IE1 plasmid.

The sacbrood virus clone (GenBank accession number MN528599) used in this study was obtained from *Apis cerana* larvae collected in Fuzhou, China. Although this isolate shares genetic similarity with Chinese variants, also known as Chinese SBV (CSBV), it exhibits only slight genetic differences compared with the typical SBV genome found in *A. mellifera* populations in the US and Europe. Moreover, this variant is prevalent in honey bee populations across East and Southeast Asia. In a previous study [26], I referred to this variant as AcSBV, but it is possible that *A. mellifera* populations may also be infected by this variant in the field [27,28]. Given the taxonomical uncertainty associated with this variant and that the main focus of this study is not the phylogenetic analysis of SBV variants, we consistently use the abbreviation “SBV” throughout the article.

**Figure 1:**
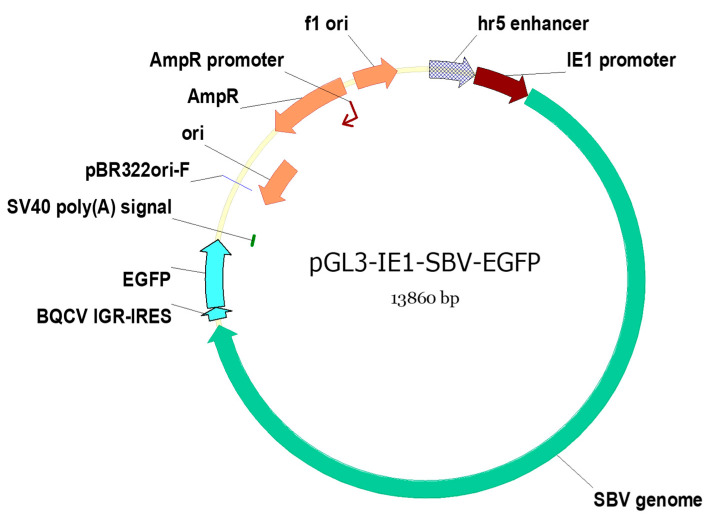
Map of the constructed plasmid pGL3-IE1-SBV-EGFP (IE1-SBV).

The SBV genome and pGL3-IE1 plasmid were amplified using PCR with the primers designed to replace the luciferase gene region on pGL3-IE1 with the SBV clone. This SBV clone utilized enhanced green fluorescence protein (EGFP) expression tag inserted within the 3′ untranslated region (UTR) [14]. The translation of EGFP is driven by a separate internal ribosome entry site (IRES) cloned from Black queen cell virus (BQCV IGR-IRES, as shown in Figure 1). The design of the EGFP-expressing tag ensures its dissociation from the cloned SBV open reading frame (ORF) as previous attempts to mutate sequences within the ORF were unsuccessful. Previously, this identical clone has been demonstrated to induce overt infections in *A. cerana* larvae, and its estimated infectious dose was determined in our previous study. [14].

The SBV clone PCR was conducted using the primer set T7-SBV5′ and SV40-EGFP-r. The pGL3-IE1 plasmid was also PCR amplified using the primer set T7-IE1-R and SV40F. These primers (listed in Table 1) included an additional overlapping region at 5′ end to facilitate Gibson assembly. Phanta Max Super-Fidelity DNA Polymerase (Vazyme, Nanjing, China) was used for the PCR, and the PCR settings and recipes followed the annotations in Table 1 and the manufacture’s suggestions, which were identical to those used in our previous study [14]. The poly(A) region of the original clone was removed since the designated pGL3-IE1 plasmid has a simian virus 40 (SV40) poly(A) signal sequence that adds poly(A) region to the transcribed RNA (Figure 1).

The pGL3-IE1-SBV-EGFP plasmid (referred to as IE1-SBV) was constructed using the Gibson assembly method [30] and the pEASY-Uni Seamless Cloning kit from Transgene. The map of the assembled plasmid is shown in Figure 1. The assembled plasmid was transfected into Stbl3 *E. coli* competent cells (Shanghai Weidi Biotechnology) using the suggested method, with the incubation temperature set at 30 °C. The transfected *E. coli* cells were cultured on LB plates with ampicillin at 30 °C for 20 h. Selected bacterial colonies were propagated in 5 mL LB medium with ampicillin at 30 °C (on an orbital shaker at 200 rpm) for 20 h. Three milliliters of the cultured *E. coli* solution was used for plasmid extraction using E.Z.N.A. Plasmid DNA Mini kit (Omega), while the remainder was mixed with glycerol (approximately 20% by volume), thoroughly mixed, and stored at −80 °C for further propagation. The extracted plasmids were linearized using EcoRI and their sizes were determined through gel electrophoresis. Plasmids of the expected size were subjected to Sanger sequencing using ABI 3730XL (performed by BioSune, Shanghai, China). The sequencings were conducted using known primers near the SBV genome termini [14] and the known primers were listed on pGL3-IE1 map (Addgene plasmid #52894) to confirm the correctness of the assembly. The *E. coli* culture containing the correct plasmid was further propagated by transferring 100 μL of the culture stored at −80 °C into 200 mL fresh LB with ampicillin. The culture was incubated at 30 °C (on an orbital shaker at 200 rpm) for 20 h. The plasmid was isolated using FastPure Endo-free Plasmid Maxi kit (Vazyme).

This plasmid is modified from the pGL3-IE1 plasmid [25] that contains IE1 promoter with hr5 enhancer to express the SBV clone, and the SV40 poly(A) signal adds poly(A) region to the transcribed SBV clone. The EGFP expressing tag is induced by a separated IGR-IRES cloned from BQCV, and it is independent of the clone infectivity [14]. Elements on the figures are as follows: f1 ori (f1 bacteriophage origin of replication); pBR322ori (origin of replication from pBR322 plasmid, resulting in approximately 15–20 copies per cell); AmpR: ampicillin resistance; SV40 poly(A) signal (Simian virus 40-derived signal that facilitates polyadenylation of transcribed RNA); and hr5 enhancer and IE1 promoter (hr5, homologous region 5, fused with the immediate-early gene (IE1) promoter, from Autographa californica multiple nucleopolyhedrovirus, AcMNPV. IE1 promoter enables transcription initiation without the requirement of other viral proteins).

### 2.3. Transfection of the IE1-SBV Plasmid in Sf9 Cells

The conventional cationic liposome method was employed to deliver the IE1-SBV plasmid DNA into cultured cells and honey bee pupae. Since a virus-free immortal cell line of honey bees was not yet widely available at the time, the commonly available insect cell line originating from *Spodoptera frugiperda*, Sf9 (Procell Life, Wuhan, China), was utilized as the in vitro host cells for IE1-SBV in serum-free culture medium, Sim SF medium (Sino-biological, Beijing, China). Lipofectamine 2000 (Invitrogen, Waltham, MA, USA) was used as the liposome reagent for the transfection. Lipofectamine 2000 has been widely used in studies, including live insect injections for RNAi trials [31].

The Sf9 cells were transfected according to the recommended protocol for adherent cells using Lipofectamine 2000. Half-density cells (4 × 10^5^) were seeded on each well of 6-well plates and transfected with 4000 ng of the IE1-SBV plasmid or pGL3-IE1 plasmid per well. The plasmid DNA was diluted into a volume of 250 μL using Sim SF medium (Sino-biological, China), with 4000 ng of plasmid DNA. This diluted plasmid DNA was then mixed with 250 μL Lipofectamine solution, prepared by combining 240 μL Sim SF with 10 μL Lipofectamine 2000. The mixture was incubated at room temperature for 20 min. Subsequently, the mixture was added to a well of 6-well plates containing seeded Sf9 cells, along with 1.5 mL Sim SF medium. Each plasmid tested underwent three repeats, with three wells allocated for each repeat.

### 2.4. Plasmid Injection in Honey Bee Pupae

To assess the ability of IE1-SBV to synthesize the clone in honey bees, we conducted injections of IE1-SBV and pGL3-IE1 (empty vector) plasmids using Lipofectamine 2000 (Invirtrogen) into honey bee pupae. A modified injection protocol was developed based on preliminary trials using Lipofectamine 2000 mixed with plasmid solutions at different concentrations. Initial tests with various concentrations of the IE1-SBV plasmid were performed in *A. mellifera* to determine the optimal amount for injection. Following the manufacturer’s manual, which suggested a ratio of 2–4 μL Lipofectamine 2000 per 1000 ng of DNA, we decided to use 4 μL Lipofectamine 2000 mixed with 4 μL of DNA plasmids, totaling 8 μL. This volume was selected to be 20% lower than the maximum capacity for pupa injection to enhance feasibility. To determine the maximum capacity, we injected PBS with blue ink into 24 pupae and observed any potential leakage and distribution of the injection within the bee pupae.

In the first trial, three groups of eight *A. mellifera* pupae were injected with 750, 1000, and 1200 ng of the IE1-SBV plasmid DNA. Among the injected pupae, only those in the groups receiving 1000 and 1200 ng of the IE1-SBV plasmid exhibited detectable virus infection. However, the infection rate was low, with only one and two out of the eight injected pupae showing infection in the RT-qPCR exam. Therefore, we continued with pupae injections and increased the amount of injected plasmid to 1800 ng in a subsequent trial using *A. mellifera* pupae in December 2021.

The injection protocol involved concentrating the plasmid using a 10 kDa centrifugal filter (0.5 mL, Millipore) and diluting it in Sim SF medium (Sino-biological, China) to the desired concentrations before mixing it with Lipofectamine 2000. The mixture was prepared in bulk for 20 pupae (160 μL in total) and incubated for at least 20 min at room temperature. Each pupa was then injected with 8 μL of the mixture, which contained 1800 ng plasmid DNA and 4 μL Lipofectamine 2000. Pupae in the white- to pink-eye stage (within 2–3 days after pupation) were carefully extracted from capped cells using a rubber air blower and soft entomology forceps. The pupae were transferred to a Petri dish in a 35 °C growth chamber with 70% relative humidity. For each injection, 12–20 pupae were taken out of the growth chamber, and a LeTkingok syringe propeller with a 1-mL disposable syringe and a 30 G (for the trial injecting 1800 ng plasmid) or 32 G (for the trial injecting 2500 ng) needle was used to inject the 8 μL plasmid mixture into each pupa over a period of 6 s. The injected pupa was then held still with the needle for an additional 10 s to allow the injected liquid to distribute evenly within the pupa and minimize post-injection leakage. Pupae that did not exhibit significant leakage after injection were transferred to a 96-well cell culture plate and returned to the 35 °C growth chamber for a 5-day incubation period. After incubation, each pupa was transferred into an Eppendorf tube containing 250 μL RNA keeper (Vazyme) and stored according to the manufacturer’s suggestions until RNA extraction.

In December 2022, we identified a few *A. cerana* colonies that had no detectable SBV infections. We conducted a similar trial by injecting different concentrations of the IE1-SBV and pGL3-IE1 plasmids (1250, 2500, 5000 ng) into *A. cerana* pupae harvested from two different *A. cerana* colonies. Each group had 16 pupae injected, and 12 pupae were selected for RT-qPCR analysis using the TaqMan method for SBV quantification. The results showed that 2500 ng resulted in the highest infection ratio among the tested concentrations. Subsequently, we conducted a parallel trial using *A. mellifera* pupae and *A. cerana* pupae from another colony. Twelve pupae were injected with 2500 ng of the IE1-SBV or pGL3-IE1 plasmid, and eight of the twelve pupae were randomly selected for the RT-qPCR examination.

### 2.5. Cell Preparation and Staining for Confocal Microscopy Analysis

To facilitate handling and observation under a confocal microscope, the Sf9 cells were seeded on cover glasses (WHB scientific, Shanghai, China) placed within 6-well plates before the transfection. The same transfection protocol using Lipofectamine 2000, as described earlier for the 6-well plates, was employed for transfecting IE1-SBV or pGL3-IE1 (original plasmid without SBV genome) plasmids. After 48 h incubation, the cover glasses were removed from the medium and rinsed twice in PBS. They were then transferred into 4% paraformaldehyde in PBS as a fixative for 15 min. Subsequently, the cover glasses were rinsed twice in PBS to remove the fixative.

To reveal nuclei, the cover glasses were stained in DAPI solution (a fluorescent DNA stain; Solar-bio, Beijing, China) followed by staining with Dil stain (a lipophilic fluorescent stain for membrane structures; Solar-bio, Beijing, China). The cover glasses were washed twice in PBS and then embedded in an anti-fade mounting medium (glycerol-based, Solar-bio, Beijing, China). Preliminary observations indicated that cells expressing EGFP fluorescence, the expressing tag of IE1-SBV plasmid, appeared to have over-developed membrane structures under visible light observation. To differentiate the membrane structures in transfected and normal cells, Dil stain was added. A low concentration of Dil stain (PBS with 0.02% of the stock solution, 1 mg/mL in DMSO) was used to prevent over-exposures.

The cells were observed using a Leica SP8 confocal microscope.

### 2.6. Plasmid-Injected Pupa Observations under a Stereo Fluorescence Microscope

To reveal the EGFP expression in honey bee pupae, the pupae were observed under a fluorescence stereo microscope (Leica M205 FA). The abdomens were dissected using a surgical scalpel, and subsequently secured in place using insect pins to reveal the pupal tissues under the Leica M205 FA microscope. After the observation, the pupa was transferred into tubes with RNA keeper (Vazyme). Only the last repetition of the IE1-SBV and pGL3-IE1 plasmid (1800 ng)-injected *A. mellifera* pupae were observed under the fluorescence stereo microscope.

### 2.7. RNA Extraction and Reverse Transcription for SBV Clone Infection Quantification

The positive strand of the SBV clone was quantified using reverse transcription quantitative polymerase chain reaction (RT-qPCR) to assess the infection intensities. For pupae, total RNA was extracted individually by homogenizing each pupa in 250 μL RNA keeper (R501, Vazyme) and subjecting 30 μL homogenate to RNA extraction using LabServ Universal RNA kit (Thermo-Fisher, Waltham, MA, USA) with a KingFisher (Thermo-Fisher) automatic manipulator, following the manufacturer’s suggestions. The extracted RNA was eluted into 30 μL RNase-free water and then reverse transcribed.

For Sf9 cells, the culture medium was removed, and the cells were rinsed with PBS twice to remove most of the medium residues. Plasmid residues were eliminated by the addition of DNaseI (Invitrogen) according to the protocol provided in the manual. After removing the liquid from the well, 1 mL of the lysis buffer from the LabServ kit (Thermo-Fisher) was added to each well of the 6-well plate, and the cells were scraped from the surface using a rubber scraper. The cell–lysate mixture was transferred into 1.5 mL Eppendorf tubes and then processed using the manufacturer’s suggested methods to extract total RNA.

We employed a two-step RT-qPCR protocol with absolute quantification to determine the SBV genome copy numbers. The same amount of RNA from Sf9 cell lysates and honey bee pupa homogenates were used for cDNA synthesis. RNA samples were quantified using Nanodrop (Thermo-Fisher) and 1000 ng of each RNA sample was used for cDNA synthesis. The cDNA was all synthesized using HiScript II Q Select kit with gDNA remover (R232 kit, Vazyme) following the manufacturer’s protocol. The qPCR was performed using the universal SBV TaqMan method [29] (primers and probe were listed in Table 1) with AceQ qPCR Probe Master Mix (Vazyme). The IE1-SBV plasmid DNA was used as the standards in absolute quantification PCR. The plasmid DNA concentration was measured using a Nanodrop (Thermo-Fisher) and copy numbers were estimated based on the molecular weight of the plasmid sequence. A standard curve was generated by serial dilution (10-fold dilution of seven concentrations) in each qPCR. The SBV copy numbers were estimated in CFX manager 3.1 (Biorad, Shanghai, China).

Upon analyzing our preliminary results, we identified the potential for IE1-SBV plasmid DNA residues within our extracted RNA to influence quantification. To mitigate this risk, we implemented a quality control process for all synthesized cDNA by employing a primer set tailored to the IE1 promoter region (Table 1). As this region is not transcribed into RNA, any presence of IE1-SBV plasmids within the cDNA renders the RNA samples and results unusable. The quality control was conducted using qPCR with SYBR green methodology, employing ChemQ Universal SYBR qPCR Master Mix (Vazyme) and 400 pM primers in a three-step program, with 95 °C for 10 s and 60 °C for 10 s, followed by 72 °C for 20 s, for 40 cycles.

### 2.8. Tagged RT-PCR to Assess Negative Strand Presence

Tagged RT-PCR was employed to investigate the presence of the negative strand in RNA samples isolated from transfected Sf9 cells and injected honey bee pupae. This evaluation allowed us to assess whether the expressed viral genome RNA was functional and capable of replications. We included two RNA samples of each group from the pupae injection that exhibited positive results and high SBV copy counts in the RT-qPCR examination for the detection of the negative strand. Additionally, all RNA samples from Sf9 cells transfected with either the IE1-SBV or pGL3-IE1 plasmid were included in the detection process. Due to the low copy numbers obtained in the quantification PCR results, a semi-nested PCR approach utilizing two sequential PCRs was applied to enhance the sensitivity and specificity of the assay. Tagged primer (Table 1) was used in cDNA synthesis using a HiScript II kit with gDNA remover (R212 kit, Vazyme) following the manufacturer’s suggested protocol, except for doubling the DNase treatment time. The cDNA samples were subjected to the first PCR using the tag primer and primer R1 (Table 1), whilst 0.5 μL cDNA was used in a 25 μL reaction using Phanta Max Super-Fidelity DNA Polymerase (Vazyme). The PCR products were diluted 10 times, and then 1 μL of the dilutions were subjected to the second PCR using the same tag primer and R2 (Table 1). A negative control, identical in all aspects except for the absence of reverse transcriptase, was conducted for each RNA sample to examine the presence of any plasmid DNA residue or unspecific detection. The PCR products were visualized using 1% agarose gel electrophoresis.

## 3. Results

Transfection Efficiency and Fluorescent Visualization of IE1-SBV and pGL3-IE1 Transfected Sf9 Cells

**Figure 2:**
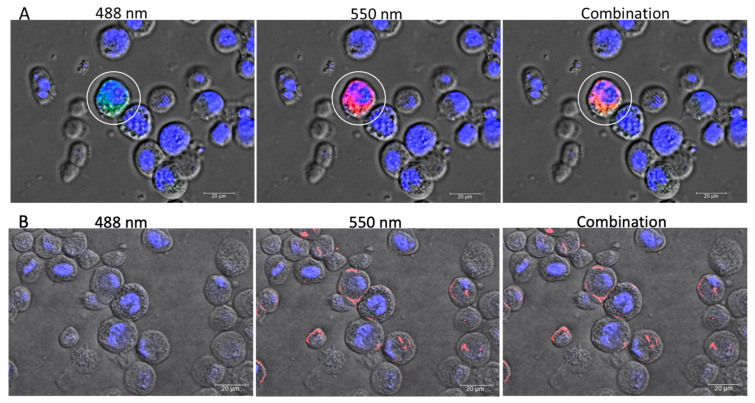
Confocal Microscopy Analysis of Transfected Sf9 Cells: IE1-SBV and pGL3-IE1. Confocal microscopy was utilized to visualize transfected Sf9 cells after 48 h incubation, indicated by white circles. (**A**) represents IE1-SBV-transfected cells, while (**B**) shows pGL3-IE1 (the original plasmid without the cloned SBV genome). Three images were captured for the same field of view for both (**A**,**B**), encompassing DAPI staining (blue, filter setting: excitation 350 nm, emission 420 nm) and visible light observation. The left image (488 nm) presents the overlapped observations with 488 nm excitation and 525 nm emission for EGFP visualization. The center image (550 nm) exhibits the overlap with 550 nm excitation and 585 nm emission for Dil-stain visualization. The right image displays the merged images of all channels. The scale bar represents 20 µm.

The transfection of the IE1-SBV plasmid using Lipofectamine 2000 resulted in a low overall transfection rate. Only a small percentage (approximately 5–10%) of the cells transfected with IE1-SBV exhibited fluorescence under 488 nm excitation, whereas no cells transfected with pGL3-IE1 showed fluorescence under 488 nm. The transfection rate of IE1-SBV was determined by calculating the proportion of cells showing fluorescence under the filter setting at 488 nm (EGFP excitation light) within each microscope view. Cells that exhibited no emission signals under 488 nm and displayed only dim fluorescence under 550 nm for the Dil stain (Figure 2A) were considered non-transfected cells. In the IE1-SBV-transfected cells, confocal observations revealed that the signals of EGFP and Dil stain were colocalized within the cytoplasm (circled by a white line in Figure 2A). In contrast, cells transfected with pGL3-IE1 (Figure 2B) did not show strong Dil stain signals within the cytoplasm, indicating that only IE1-SBV transfection led to significant staining.

### 3.1. Exploration of Fluorescence in IE1-SBV-Injected A. mellifera Pupae and Comparison with Controls

**Figure 3:**
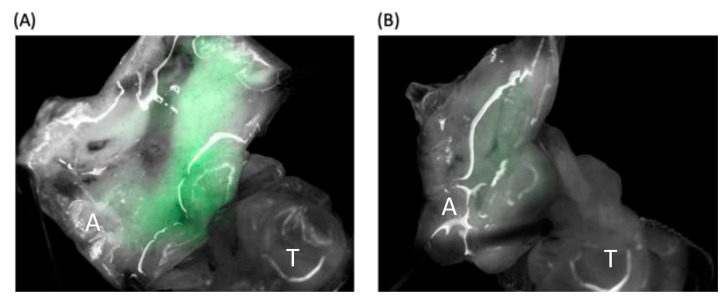
Evaluation of EGFP Expression in Dissected Pupae. The evaluation of EGFP (Enhanced Green Fluorescent Protein) expression levels in dissected *A. mellifera* pupae using a stereo fluorescence microscope after five days incubation. The green fluorescence was more intense in IE1-SBV-injected pupae (**A**) compared with controls (**B**), where the green fluorescence was also detected, possibly due to auto-fluorescence under the microscope. The photos were processed and overlapped using ImageJ with identical settings. In the figures, the indicators “T” and “A” correspond to the thorax and abdomen of the pupa, respectively.

The IE1-SBV- and pGL3-IE1-injected *A. mellifera* pupae were observed under a fluorescence stereo microscope using visible light and UV light with a filter setting for EGFP observation (470 nm excitation and 525 nm emission). For the observation, we selected the last repetition of the 1800 ng injection of *A. mellifera* pupae. Initially, we directly observed the intact pupae and noted that three out of the seven IE1-SBV-injected pupae exhibited relatively stronger fluorescence under the microscope, although it was not distinctly visible under the excitation light. The presence of autofluorescence from pupal tissues and the cuticle posed challenges in differentiating the fluorescence signal. To overcome this, we opened the abdomen of the pupae using a surgical scalpel and immobilized them with insect pins under the microscope for better exposure to the excitation light (Figure 3). Figure 3A depicts an IE1-SBV-transfected pupa with stronger fluorescence in the developing tissues within the abdomen, in comparison with the control (Figure 3B). Although the fluorescence intensity of the pupa in Figure 3A was distinguishable using image analysis software, the autofluorescence of the pupal tissues in Figure 3B made it difficult to clearly discern them during the observation. Figure 3A displayed the strongest fluorescence among the three that were suspected as infected by the clone and expressed EGFP during the observation. Furthermore, out of the three suspected pupae in the IE1-SBV group, only two were found to have detectable SBV in the subsequent RT-qPCR.

### 3.2. Quantifications of SBV Genome Copies in Transfected Sf9 Cells and Honey Bee Pupae

**Figure 4:**
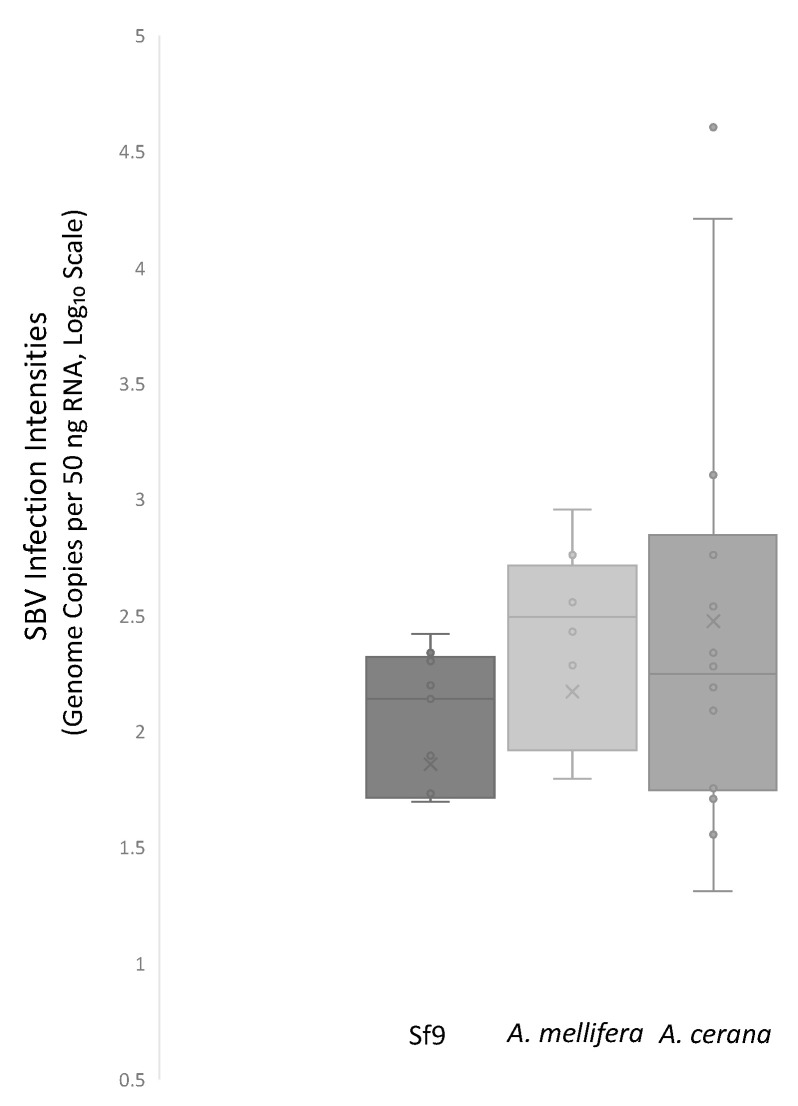
Quantitative PCR Results of SBV Genome Copies in cDNA Samples from Sf9 Transfected by IE1-SBV Plasmid and IE1-SBV-Injected Honey Bee Pupae.

RNA samples isolated from Sf9 cells transfected with the IE1-SBV plasmids exhibited detectable levels of SBV viral RNA, whereas no SBV viral RNA was detected in cells transfected with pGL3-IE1. The two-step RT-qPCR with absolute quantification method revealed an average of 145 ± 28.3 genome copies (Figure 4) of SBV per 50 ng of RNA in cells transfected with IE1-SBV. All cDNA samples included in the RT-qPCR passed the quality control examination for IE1-SBV plasmid residues, as confirmed by a primer set design based on the IE1 promoter region.

An injection of the IE1-SBV resulted in detectable infections within honey bee pupae, and the quantification of the IE1-SBV plasmid-injected pupae demonstrated higher average SBV genome counts compared with Sf9 cells. However, the standard deviations were higher in pupae (Figure 4; raw data listed in Table S1). Two injection trials were conducted at different time points, utilizing varying plasmid quantities (1800 and 2500 ng) and involving different species of bees. In the initial trial, no *A. cerana* colony free of detectable SBV was identified, resulting in the use of only *A. mellifera* in the 1800 ng injection trial involving both IE1-SBV and pGL3-IE1 plasmids. Despite the intended inclusion of eight surviving pupae in each group for the three repetitions, the actual numbers fell short, likely due to high mortalities following injection (Table S1). Only 15 out of the 24 IE1-SBV-injected pupae survived the incubation period. The detected SBV genome copies ranged from 1.91 to 8.96 × 10^2^ (with an average of 4.18 × 10^2^ ± 1.24 × 10^2^ per 50 ng RNA) in the 1800 ng IE1-SBV-injected *A. mellifera* pupae, resulting in an overall infection ratio of 33% (five out of the fifteen injected pupae). The single positive pupa in the pGL3-IE1 group exhibited 3.36E02 SBV genome copies. Due to the relatively low infection rates observed in the 1800 ng injection, another trial was conducted using higher plasmid concentrations injected into *A. cerana* pupae after the identification of *A. cerana* colonies free of detectable SBV in December 2022. The results indicated a 75% infection rate in the group injected with 2500 ng of the IE1-SBV plasmid, while the group injected with 5000 ng plasmid resulted in a 50% infection rate (Table S1). This difference could potentially be attributed to the identical amount of Lipofectamine (4 μL) being injected alongside the plasmid. The SBV quantification in the 2500 and 5000 ng injection groups yielded similar results, leading to another 2500 ng injection trial using *A. cerana* pupae from another colony, with *A. mellifera* pupae injected in parallel for comparison. In this repetition, the 2500 ng IE1-SBV injection resulted in a 75% infection rate in *A. cerana* (six out of the eight injected pupae) and a 25% infection rate in *A. mellifera* (two out of the eight injected pupae). The 1800 and 2500 ng plasmid-injected *A. mellifera* pupae exhibited similar SBV counts (Table S1), with the data presented in the same box plot in Figure 4. The SBV qPCR results of *A. cerana* and *A. mellifera* pupae showed similarities; however, there were highly infected pupae observed in the *A. cerana* group (Figure 4). The pGL3-IE1-injected *A. mellifera* and *A. cerana* pupae were all free of detectable SBV in the second trial.

The figure illustrates the SBV genome copies detected in cDNA samples obtained from Sf9 cells transfected with the IE1-SBV plasmid (48 h incubation) and honey bee pupae injected with IE1-SBV (five days incubation). Copy numbers were determined using the TaqMan method with absolute quantification, referencing IE1-SBV plasmid standards. The values on the graph represent SBV genome copies in cDNA generated from 50 ng of RNA. The Y-axis is presented in a base-10 logarithm (Log₁₀) format. Notably, the chart includes two highly infected *A. cerana* pupae: one from the first repetition displayed 40,000 genome copies, while another from the second repetition had 16,200 genome copies. Each qPCR reaction demonstrated a PCR efficiency between 90 and 100%.

### 3.3. Negative Strand Detection in Sf9 Cells and Honey Bee Pupae

**Figure 5:**
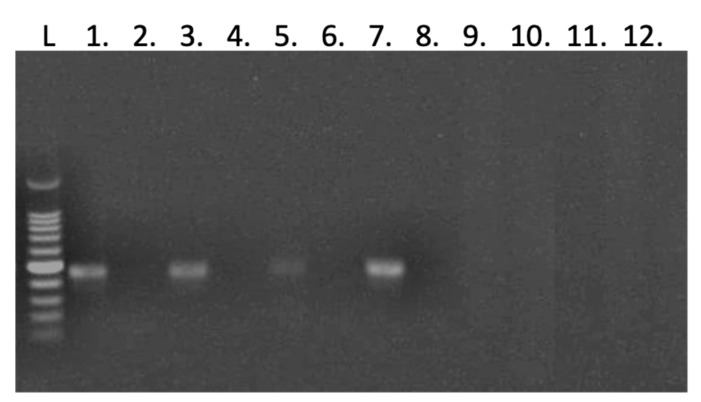
Negative Strand Detection in Plasmid-Injected Pupae. This figure displays the results of the negative strand detection assay in the pupae that received plasmid injections after five days incubation. The odd-numbered samples represent pupa RNA samples processed with reverse transcriptase, while the subsequent even-numbered samples correspond to the same samples processed without reverse transcriptase, serving as negative controls. Samples 1 to 4 correspond to *A. cerana* pupae injected with the IE1-SBV plasmid, while samples 5 to 8 represent *A. mellifera* pupae injected with the IE1-SBV. Additionally, samples 9 to 12 depict one *A. cerana* and one *A. mellifera* pupa, respectively, both injected with the pGL3-IE1 plasmid. L indicates a DNA ladder, a 100 bp DNA ladder from Transgene (Beijin).

Figure 5 presents the results of the negative strand detection in the pupa RNA samples. Both *A. mellifera* and *A. cerana* pupae injected with the IE1-SBV plasmid exhibited detectable negative strands. The negative controls, which underwent the same processes except for the addition of reverse transcriptase, did not show any detectable negative strand. Additionally, we randomly selected RNA samples from two pupae injected with the pGL3-IE1 plasmid to be included in the same detection, and the results indicated the absence of a detectable negative strand. Similar results were observed in the IE1-SBV-transfected Sf9 cells (Figure S1), where all the IE1-SBV-transfected cells showed detectable negative strands of SBV, while none of the negative controls (without reverse transcriptase) or the pGL3-IE1 plasmid-transfected cells exhibited a detectable negative strand. Three independent repeats of the IE1-SBV-transfected Sf9 cells, each comprising three technical replicates, consistently yielded identical results.

## 4. Discussion

The SBV clones were synthesized using DNA plasmids with the IE1 promoter in Sf9 cell lines and honey bee pupae. In our previous study, we demonstrated that this identical clone caused overt infection and allowed for the estimation of infection doses in *A. cerana* larvae [14]. Although Sf9 cells and the pupal stage of *A. mellifera* and *A. cerana* are not the typical hosts for overt SBV infection, they offer convenience for laboratory manipulation and maintenance. Intriguingly, our previous trial involved the injection of transcribed viral genome RNA from the identical clone into *A. mellifera* pupae [14] and yielded infection ratios comparable to those observed in the injection of the IE1-SBV plasmid in *A. mellifera* pupae. Additionally, the presence of the negative strand of the clone was consistently detected in the IE1-SBV-transfected Sf9 cells and injected pupae of both *A. mellifera* and *A. cerana*, suggesting the active replication of the cloned virus in these hosts. Although the possibility of false-positive results in the negative strand detection cannot be completely ruled out [32], our repeated transfections of Sf9 cells consistently yielded the same findings, indicating active replication within the cells. Moreover, the use of DNA plasmids with the IE1 promoter streamlines the sequence editing processes of bee-infecting RNA viruses and offers Sf9 cells and similar cell lines as valuable tools for evaluating the viability of edited clones, reducing the need for repetitive sequence editing and bioassays using honey bees. The synthesis of bee-infecting RNA virus clones using the IE1 promoter holds potential for facilitating reverse genetic studies, thereby enhancing our understanding of bee-infecting RNA virus biology and pathology.

A few limitations of using Sf9 cells, not the natural host, as a surrogate host for SBV have been revealed. Previous studies [21,33], which used purified virions and viral genome RNA, respectively, suggested the potential of SBV to infect surrogate cell lines. However, these studies reported failures to inoculate naïve cells upon subculture and similar findings were observed in a study on Deformed wing virus in Sf9 cells [34]. In this study, we also noted that the cells with the expressing tag of the SBV clone are not increased with a prolonged incubation time. Since the expressing tag is stable within the SBV clone [14], this observation suggested that the clone cannot disseminate to adjacent cells naturally or form infective virions. This phenomenon also explained the low count in the RT-qPCR for SBV genome copies of the transfected Sf9 cells, because only the cells transfected with the plasmid synthesized the SBV viral RNA. In contrast, an Iflavirus found in lepidopteran, *Perina nuda* virus, PnV [35], achieved a nearly 100% infection rate in Sf9 cells and produced millions of viruses. While Sf9 cells may not be ideal hosts for massive cloned virus production in bee-infecting RNA viruses, they can still be valuable for assessing whether the clone is capable of replicating, which is a crucial step in trials involving honey bees. By utilizing this approach, the need for extensive resources when testing multiple mutated clones simultaneously can be significantly reduced.

The confocal microscope observations of the transfected Sf9 cells illustrated obvious alterations in membrane structures within the cytoplasm. This result is consistent with a lipidomic study using *A. cerana* larvae [36], which found SBV infection significantly altered the host lipid profiles. Since the synthesized SBV clones did not disseminate to naïve Sf9 cells, these membrane structure alterations were probably not caused by the entry of SBV, which may involve an endocytosis [37]. Additional TEM observation (Figure S2) suggested these membrane structures are mitochondria, ER-like vesicles, and multi-membrane vesicles. However, based on the current results, we cannot determine if the membrane structures are involved in the antiviral responses of the cell [38,39] or intermediate vesicles forming extracellular vesicles [40,41].

The transfection method for the plasmid containing the viral full-length cDNA genome has not been fully optimized in this study. The transfection rate of the plasmids in Sf9 cells was found to be lower than 10%, which did not meet our initial expectations. However, this estimation might be an overestimate, as we only counted areas containing cells with fluorescence. Prior experiences with Lipofectamine-mediated transfections of bacmids containing a baculovirus genome of over 100 kb in size into Sf9 cells were successful; however, those experiments involved enrichment through subcultures, a process not feasible for the infectious clone in this study. Furthermore, the suggested volumes on the Lipofectamine manufacturer’s protocol were optimized for cell cultures using large volumes—specifically, 500 microliters with 4000 ng plasmids for half a million seeded cells. This suggested volume is unsuitable for honey bee pupa injection, as a honey bee pupa consists of far more cells but can only accommodate approximately 10 microliters of injection before the body cavity becomes severely distended. Consequently, we had to use much higher concentrated plasmids (1800 and 2500 ng in eight microliters final mixture) mixed with Lipofectamine. The high concentrations of plasmid DNA and liposomes may have affected their interaction, and we found that injecting 5000 ng of the plasmid did not yield more infection when the Lipofectamine was not increased. However, our application and optimization of the transfection and injection method were limited by our lack of expertise in such protocols. Exploring other transfection reagents or methods that are suitable for delivering large DNA fragments within relatively small volumes may enhance the results in cells and honey bees. Additionally, the choice of the expressing tag, EGFP, may not be the most suitable option for honey bee pupae due to the confusion with autofluorescence from the control pupae. Other alternatives, such as mCherry or nanoluc [42], may prove more effective for pupa studies.

The observed infection rate differences in pupae may be attributed to their anti-virus immune responses. The consistently low infection rates in Sf9 cells during transfection suggest that only a small number of cells within the injected pupae were successfully transfected by the plasmid, leading to the synthesis of infectious clones in limited quantities that may not have been sufficient to overcome the host’s anti-virus defenses. The results of plasmid injections in both *A. mellifera* and *A. cerana* pupae support the hypothesis that host immune responses may restrict the infection success of the injected clones. *A. cerana* pupae consistently displayed a 75% infection rate with 2500 ng plasmid in repetition trials using pupae from different colonies, while *A. mellifera* showed only a 25% infection rate with the same dosage. Similarly, another previous injection of *A. mellifera* using 1800 ng plasmid yielded a comparable infection rate (33%) in trials. A direct comparison between *A. cerana* and *A. mellifera* injections indicates that *A. cerana* is approximately three times more susceptible to the SBV clone. This finding gains further significance from the fact that *A. cerana* colonies exhibited a higher susceptibility to SBV in the field [43]. Interestingly, the observation of highly infected individual pupae was specific to the *A. cerana* pupae, suggesting some individuals in the colony were highly vulnerable to the virus. Notably, these *A. cerana* colonies had previous pre-exposures to the SBV variant during our continuous survey, which may have resulted in primed immune responses to the SBV variant and could explain the high deviations of the infection intensities; however, their susceptibility remained much higher than that of *A. mellifera*. The identified susceptibility difference between *A. cerana* and *A. mellifera* in this study necessitates further investigation with larger sample sizes, well-defined genetic backgrounds, and pre-exposures to the virus. Additional research is needed to better understand the underlying factors contributing to the differential susceptibility of *A. cerana* and *A. mellifera* to the SBV variant.

Our successful generation of infectious clones in Sf9 cells and honey bee pupae offers new possibilities for testing the viability and virulence of edited clones, reducing the time and labor involved in these critical investigations. Our approach presents an alternative in situations where bees free of the cloned virus species are unavailable in the field, as cultured cells can serve as surrogate hosts. As we consider future applications, the use of honey bee cell lines [44] or primary cultures [45] may be explored since the same plasmid can drive the clone synthesis in honey bee pupae. However, caution should be exercised regarding potential virus contamination concerns in these cultures. Continued research into the membrane structures observed in Sf9 cell cytoplasm and an exploration of alternative expressing tags for pupae studies will undoubtedly advance our understanding of bee-infecting RNA virus dynamics.

In conclusion, our study has successfully demonstrated the synthesis of SBV clones using the IE1 promoter in both Sf9 cells and honey bee pupae. Despite some limitations in the transfection method and the challenges posed by hosts, our findings hold promise for streamlining reverse genetic studies of bee-infecting RNA viruses. By utilizing DNA plasmids with the IE1 promoter, we have achieved the efficient expression and replication of the cloned virus in laboratory-controlled settings. These advancements offer a valuable tool for investigating the biology and pathology of bee-infecting RNA viruses in a more efficient and controlled manner. Overall, this study offers significant potential to advance our knowledge of bee–virus interactions and may ultimately contribute to the development of effective strategies for safeguarding the health of these critical pollinators. We hope that our work will inspire further research in this area and contribute to the collective efforts aimed at securing a healthy future for these essential pollinators.

## Figures and Tables

**Table 1:** Primers Used in This Study.

	Sequence	PCR Temp. *	Annotation
T7-SBV5′	TAA TAC GAC TCA CTA TAG TAC GAA TC	54	Purified plasmid (1 ng) was used as the template
SV40-EGFP-r	tatcttatcatgtctCTT GTA CAG CTC GTC CAT GCC		
T7-IE1-R	tatagtgagtcgtattaGTC ACT TGG TTG TTC ACG AT	54	Purified plasmid (1 ng) was used as the template
SV40F	AGACATGATAAGATACATTGATGAGTTTG		
EBV Reverse	GTGGTTTGTCCAAACTCATC		Sequencing primer
IE1-fseq	GTTATCGTGTTCGCCATTAGG		Sequencing primer
Tagged primer	aacggtcatggtggcgaataaGCAACGAAAATGAGCAACC		Negative strand detections using Tagged RT-PCR with semi-nested PCR
Tag primer	CGGTCATGGTGGCGAATAA	60	
R1	CATTGTCCACCGCACCATTA		
R2	TTCCATAGCAGCCTTCGC	58	
IE1-F	CTCCTCGTGTTCCGTTCAAG	60	Plasmid residue screening
IE1-R	CATCCGCCGACATACAATG		
SBV374F	CAG TGG ACT CTT ATA CCG ATT TG	60	TaqMan for SBV detection, detailed recipe was listed in [29].
SBV551Rd	GAG GTA ATA ACT TTT CGC CAY ACT A		
SBV469F (probe)	FAM-GAC GAA GAA TCT GGA ATG T-MGB		

* Except for TaqMan, reversed transcription, and sequencing, the mentioned temperature represents the annealing temperature used in PCR with Phanta Max Super-Fidelity DNA Polymerase (Vazyme). The remaining PCR recipe and program settings followed the manufacture’s manual.

## Data Availability

Not applicable.

## Supplementary Materials

The following supporting information can be downloaded at: https://www.mdpi.com/article/10.3390/v15091866/s1, Figure S1: The negative strand examination performed using Tagged RT-PCR in Sf9 cells; Figure S2: Conventional transmission electronic microscope (TEM) observations of the IE1-SBV transfected Sf9 cells; Table S1: Results of the pupa injection trials.
