# Supplementary material for: A DNA Plasmid-Based Approach for Efficient Synthesis of Sacbrood Virus Infectious Clones within Host Cells
Source: Viruses. 2023 Sep 1;15(9):1866. doi: 10.3390/v15091866 (PMC10537335; doi:10.3390/v15091866)
Supplement: Supplementary file 1 [file viruses-15-01866-s001.zip › viruses-2545263-supplementary.pdf]

Table S1. Results of the pupa injection trials.

Colony	Group	Injected Plasmid	Tested Pupae	SBV Detected	Quantification results (SBV copies in 50 ng RNA)
1	*A. mellifera* (1800 ng)	IE1-SBV	4	0	
1	*A. mellifera* (1800 ng)	pGL3-IE1	5	0	
2	*A. mellifera* (1800 ng)	IE1-SBV	4	3	1.91E+02; 2.68E+02; 3.60E+02
2	*A. mellifera* (1800 ng)	pGL3-IE1	5	1	3.36E+02
3	*A. mellifera* (1800 ng)	IE1-SBV	7	2	3.77E+02; 8.96E+02
3	*A. mellifera* (1800 ng)	pGL3-IE1	6	0	
4	*A. cerana* (1250 ng)	IE1-SBV	12*	1	255.6
4	*A. cerana* (2500 ng)	IE1-SBV	12*	8	56.8; 4.0E+04; 20.36; 2.17E+02; 3.60E+01; 3.45E+02; 5.12E+01; 1.23E+02
4	*A. cerana* (5000 ng)	IE1-SBV	12*	6	1.70E+02; 6.92E+01; 7.24E+01; 3.31E+03; 2.40E+02; 2.08E+02
4	*A. cerana* (2500 ng)	pGL3-IE1	12*	0	
5	*A. mellifera* (2500 ng)	IE1-SBV	8*	2	5.76E+02; 6.20E+01
5	*A. mellifera* (2500 ng)	pGL3-IE1	8*	0	
6	*A. cerana* (2500 ng)	IE1-SBV	8*	6	190.4; 1.28E+03; 5.76E+02; 1.62E+02; 1.62E+04; 1.54E+02
6	*A. cerana* (2500 ng)	pGL3-IE1	8*	0	

* Indicating the number selected for RT-qPCR, not the number survived through the incubation.

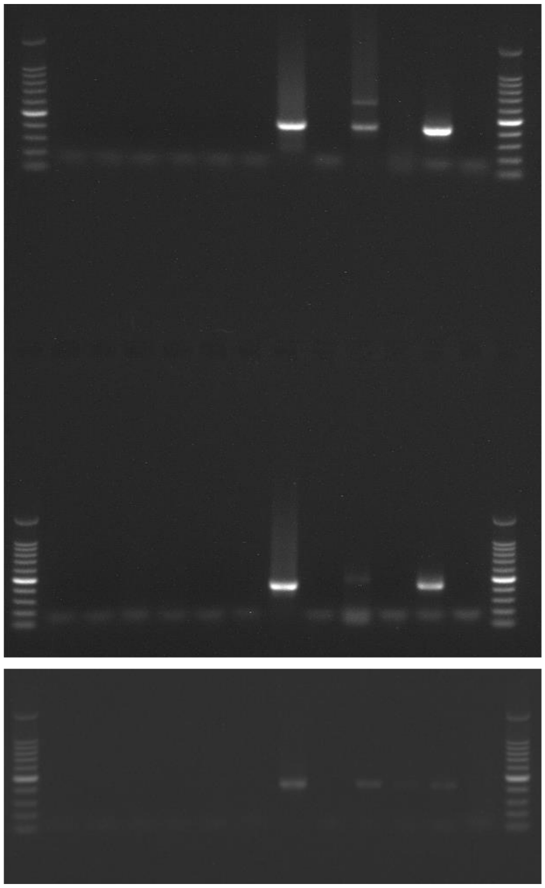

Fig. S1. The negative strand examination was performed using Tagged RT-PCR. Three biological replicates were conducted using different cultures of Sf9 cells. In each replicate, three technical replicates were performed for the IE1-SBV and pGL3-IE1 (empty vector). Six RNA samples were collected in each biological replicate, and a negative control was included in which reverse transcriptase (RTase) was omitted from the reaction. The samples were arranged from left to right, starting with three control samples (with and without RTase), followed by three IE1-SBV transfected samples.

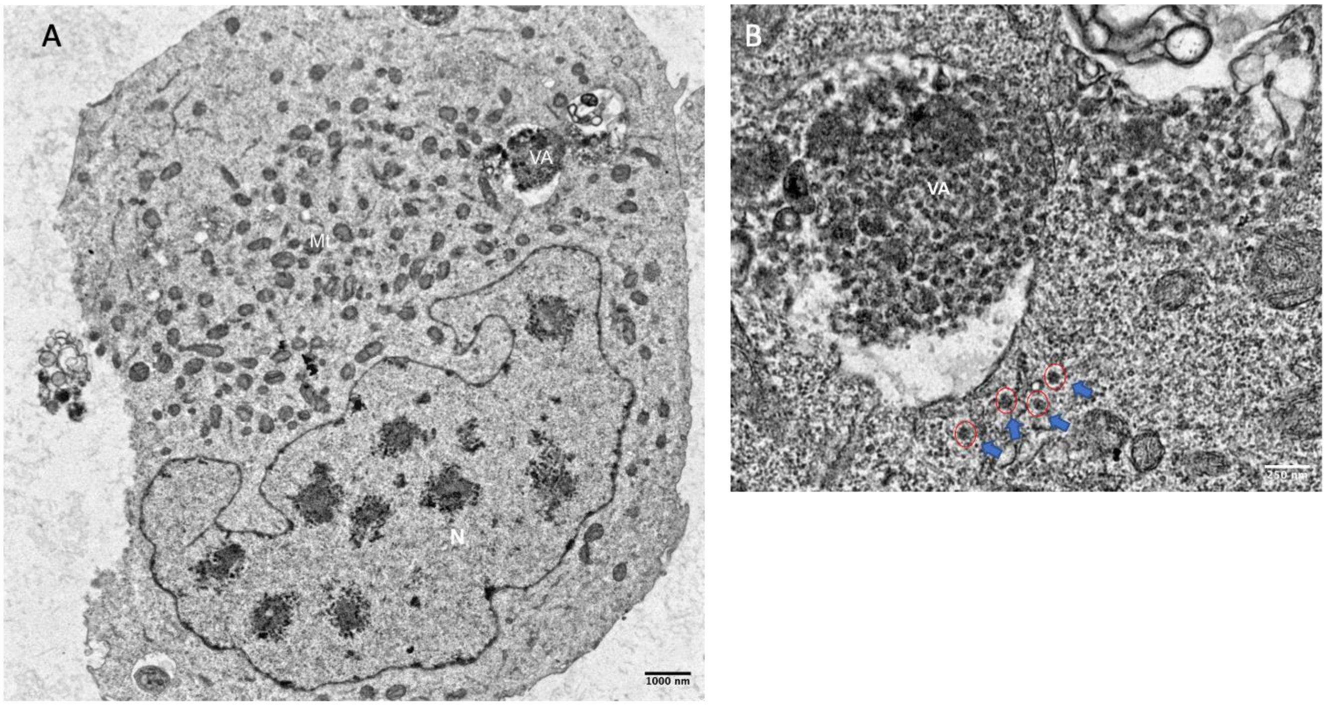

Figure S2. Conventional transmission electronic microscope (TEM) observations of the IE1-SBV transfected Sf9 cells.

TEM observations were conducted to investigate SBV clones' typical virion morphology and to clarify the increased membrane structures within transfected Sf9 cells. The transfected cells were fixed with 4% glutaraldehyde, centrifuged, and kept in a 4˚C fridge for 24 hours. After fixation, the samples underwent PBS washes, treatment with 1% uranyl acetate, and dehydration using a series of ethanol concentrations. They were then embedded using SPI-Chem Spurr low viscosity kit and transferred into a series of resin/acetone mixtures. The samples were solidified in molds with a temperature gradient and were then sliced using a diamond knife on Leica EM UC7 Ultramicrotome and observed on a Hitachi HT-7700. The acquired images were processed using FIJI (ImageJ).

(A) The image shows an IE1-SBV transfected Sf9 cell. A specific region near the virus-related aggregation was selected for closer examination, as shown in (B). Aggregated particles exhibiting SBV morphological features (average diameter of 42.76nm, estimated from those that have distinct

edges) were mainly localized within vesicles, which displayed multi-membrane structures. Apart from the virus-related aggregations, individual putative virus particles (circled in red line and indicated by arrows) were also observed scattered throughout the cytoplasm, with many attached to endoplasmic reticulum vesicles. Abbreviations used in the figures are as follows: Mt (mitochondria), N (cell nucleus), and VA (virus-related aggregation).
